# Hypoxia-preconditioned adipose-derived stem cells combined with scaffold promote urethral reconstruction by upregulation of angiogenesis and glycolysis

**DOI:** 10.1186/s13287-020-02052-4

**Published:** 2020-12-11

**Authors:** Xiang Wan, Min-kai Xie, Huan Xu, Zi-wei Wei, Hai-jun Yao, Zhong Wang, Da-chao Zheng

**Affiliations:** Department of Urology, Shanghai Ninth People’s Hospital, Shanghai Jiao Tong University School of Medicine, No.639, Zhizaoju Road in Huangpu District, Shanghai, 200011 China

**Keywords:** Hypoxia, Adipose-derived stem cells, Angiogenesis, Glycolysis, Urethral, Tissue engineering

## Abstract

**Rationale:**

Tissue engineering is a promising alternative for urethral reconstruction, and adipose-derived stem cells (ADSCs) are widely used as seeding cells. Hypoxia preconditioning can significantly enhance the therapeutic effects of ADSCs. The low oxygen tension of postoperative wound healing is inevitable and may facilitate the nutritional function of ADSCs. This study aimed to investigate if hypoxia-preconditioned ADSCs, compared to normoxia-preconditioned ADSCs, combined with scaffold could better promote urethral reconstruction and exploring the underlying mechanism.

**Methods:**

In vitro, paracrine cytokines and secretomes that were secreted by hypoxia- or normoxia-preconditioned ADSCs were added to cultures of human umbilical vein endothelial cells (HUVECs) to measure their functions. In vivo, hypoxia- or normoxia-preconditioned ADSCs were seeded on a porous nanofibrous scaffold for urethral repair on a defect model in rabbits.

**Results:**

The in vitro results showed that hypoxia could enhance the secretion of VEGFA by ADSCs, and hypoxia-preconditioned ADSCs could enhance the viability, proliferation, migration, angiogenesis, and glycolysis of HUVECs (*p* < 0.05). After silencing VEGFA, angiogenesis and glycolysis were significantly inhibited (*p* < 0.05). The in vivo results showed that compared to normoxia-preconditioned ADSCs, hypoxia-preconditioned ADSCs combined with scaffolds led to a larger urethral lumen diameter, preserved urethral morphology, and enhanced angiogenesis (*p* < 0.05).

**Conclusions:**

Hypoxia preconditioning of ADSCs combined with scaffold could better promote urethral reconstruction by upregulating angiogenesis and glycolysis. Hypoxia-preconditioned ADSCs combined with novel scaffold may provide a promising alternative treatment for urethral reconstruction.

## Introduction

A variety of diseases, such as congenital diseases, malignancies, and trauma, can lead to anterior urethral defects or strictures, which severely influence the physical condition and quality of life of patients [1]. Buccal mucosa and penile skin are the most popular tissues used to repair urethral defects in the clinic. However, donor site morbidity, limited supply of materials, and complications, such as urethrocutaneous fistula and restenosis, hinder the further clinical application of these tissues [2]. Many studies have demonstrated that cell-seeded biomaterials might be an effective method and show great promise in urethral reconstruction [3, 4].

Adipose-derived stem cells (ADSCs) are widely used in regenerative medicine [5]. Increasing evidence has demonstrated that trophic and paracrine functions, rather than multipotency, account for the therapeutic benefits of ADSCs [6]. Various growth factors and bioactive cytokines are released to stimulate the growth of nearby cells by paracrine signaling [7, 8]. For this reason, recent studies have focused on how to increase the paracrine activities of stem cells to improve angiogenesis and blood flow recovery for tissue regeneration [9, 10].

On the one hand, ADSCs are more adapted to hypoxia, and under hypoxia preconditioning, these cells can enhance their paracrine activity, proliferation, and survival [11, 12]. ADSCs reside in an environment with a relatively low oxygen level between 1 and 5%, which is significantly lower than the oxygen level required by other cells (i.e., 20–21% O_2_) [13]. For example, previous studies in a hindlimb ischemia model showed that adapting ADSCs by hypoxia preconditioning improves angiogenesis, accelerates tissue regeneration, and improves blood perfusion [14]. On the other hand, postoperative ischemia and hypoxia are inevitable and may lead to poor prognosis [15]. The microenvironment of the early period of tissue damage, wound recovery, or tissue engineering repair is characterized by ischemia and low oxygen tension [16]. Compared to other cells, ADSCs that reside in “naturally hypoxic conditions” could better survive and adapt to this condition and secrete more paracrine therapeutic factors, including vascular endothelial growth factor-A (VEGF-A). It is plausible that this characteristic of stem cells may be applied to repairing damaged tissue and facilitating tissue regeneration [17, 18].

Angiogenesis plays a significant role in fracture healing, tissue regeneration, and tissue engineering [19]. Angiogenesis provides nutrients, oxygen, and growth factors and removes metabolic waste. The formation of vessels is inextricably linked with the metabolic state [20]. Interestingly, vessel endothelial cells are highly dependent on glycolysis rather than oxidative phosphorylation; approximately 90% of adenosine triphosphate (ATP) in these cells is generated by glycolysis [21]. Vessel endothelial cells are highly dependent on glycolysis for ATP production, and the inhibition of glycolytic activators in vessel endothelial cells leads to vascular hypobranching [22]. VEGF is one of the most important cytokines for angiogenesis that is secreted by hypoxia-preconditioned ADSCs, and it exerts a significant influence on endothelial cells during vascularization [23]. Hypoxia-preconditioned ADSCs secreted more VEGF, as determined by enzyme-linked immunosorbent assay (ELISA) assay [17].

In our previous study, we fabricated a porous bilayered nanofibrous scaffold using a PLLA/PCL/PLGA blend. This heterogeneous, porous, bilayered, nanofibrous graft closely mimics the microstructure of the extracellular matrix (ECM) and has two sides: the inner surface with a small pore size can prevent the infiltration of urine to internal cells or transplanted cells, and it can also facilitate the confluence of urethral epithelial cells on the lumen; the outer surface with a larger pore size provides adequate space for the infiltration and regeneration of blood vessel cells and smooth muscle cells [24–26].

The underlying mechanism by which ADSCs facilitate angiogenesis is not currently completely understood [27]. We hypothesized that hypoxia-preconditioned ADSCs could secrete more paracrine cytokines, such as VEGF, to enhance the proliferation of vessel endothelial cells by upregulating glycolysis during urethral reconstruction. To address this hypothesis, in an in vitro study, we used paracrine cytokines and secretomes that were secreted by hypoxia- or normoxia-preconditioned ADSCs to treat human umbilical vein endothelial cells (HUVECs), and we observed the viability, proliferation, migration, angiogenesis, and metabolic state of the HUVECs. In an in vivo study, we evaluated the ability of hypoxia- or normoxia-preconditioned ADSCs that were transplanted onto our bilayered nanofibrous scaffold to repair urethral defects in a rabbit model by hematoxylin and eosin (H&E) staining, immunofluorescence staining, and urethrography.

## Materials and methods

### Ethical approval

All animal procedures were approved by the Animal Ethics Committee of Shanghai University of Traditional Chinese Medicine (Reference no: P2SHUTCM200424005) and carried out in accordance with the guidelines for the National Institutes of Health Guide for the Care and Use of Laboratory Animals.

### Cell isolation and culture

ADSCs were separated from inguinal adipose tissue. The 15 rabbits (2.5–3.5 kg) were subjected to general anesthesia by intravenous injection of sodium pentobarbital. The adipose tissues were isolated from the bilateral inguinal region followed by rinsing with 0.25% chloromycetin in sterilized PBS. The tissue was homogenized and digested in 50 ml 0.1% type IV collagenase (S1745401, Nordmark Biochemicals, Germany) for 50 min at 37 °C with sustained oscillations. After digestion, the solution was centrifuged at 800 rpm for 10 min. The supernatant was discarded, and the precipitate was mixed with sterilized PBS. After filtration with a cell strainer (Falcon, USA), the mixture was centrifuged at 1000 rpm for 5 min. After discarding the supernatant, the precipitate was resuspended in α-modified Eagle’s medium (α-MEM) (HyClone, Logan, UT, USA) supplemented with 10% fetal bovine serum (FBS, Gibco) and 1% Antibiotic-Antimycotic (Gibco, USA) in a 10-cm cell culture dish. The cell culture dishes were placed in a humidified incubator at 37 °C with 5% carbon dioxide. We changed the culture medium every 48 h and passaged the ADSCs when they reached 80% confluence. The 15 rabbits were used in subsequent experiments. The isolated stem cells are for each rabbit’s own use. HUVECs were separated from the umbilical cord and cultured in endothelial cell medium (ScienCell, USA) supplemented with 10% FBS (Gibco, USA) and 1% penicillin and streptomycin (Gibco, USA). The ADSCs and HUVECs were cultured in a humidified incubator with 5% CO_2_ at 37 °C.

### Identification of ADSCs

The identification of ADSCs was conducted by evaluating their adipogenic, osteogenic, and chondrogenic differentiation potential. The adipogenic differentiation, osteogenic differentiation, and chondrogenic differentiation of the ADSCs were conducted strictly in accordance with the manufacturer’s instructions (Cyagen, USA). The results of adipogenesis, chondrogenesis, and osteogenesis were identified by Oil Red O, Alizarin Red, and Alcian Blue staining (Cyagen, USA).

### Hypoxic cell culture

When ADSCs at passage 3 reached 70% confluence, the 10-cm plate was washed with sterilized PBS, and 10 ml α-MEM without FBS was added. The plates were cultured under normoxic (21% O_2_) or hypoxic (1% O_2_) conditions for 2 days. After filtration, the conditioned medium was added to a centrifugal filter unit with a 3-kDa cutoff (Millipore, Bedford, MA, USA). The centrifugal filters were centrifuged at 3000 rpm to concentrate the medium by 50-fold. The concentrated hypoxia- or normoxia-conditioned medium was mixed with FBS-free endothelial cell medium at a ratio of 1:3 and was labeled as solution H or solution N.

### Cell proliferation assays

The effects of secreted fractions from hypoxia- or normoxia-preconditioned ADSCs on the proliferation of the HUVECs were tested by a Cell Counting Kit-8 (CCK-8; Dojindo, Kumamoto, Japan) according to the manufacturer’s instructions. Briefly, 1000 HUVECs/well were seeded in a 96-well plate. The HUVECs were cultured by solution H or N and labeled as group H and N. At 0, 12, 24, and 48 h of culture, 10% CCK-8 reagent was mixed with medium and added to each well. The 96-well plate was incubated at 37 °C for 1 h. The relative number of cells was measured by absorbance at 450 nm using a microplate reader (BioTek, USA).

### Colony formation assays

The HUVECs were seeded in a 6-well plate at 500 cells/well. The cells were treated with solution H or N and were labeled as group H and N. The medium was changed every 3 days. Fourteen days later, the cells were fixed with 4% paraformaldehyde and stained with crystal violet solution for 6–10 min. Groups consisting of 50 or more cells were scored as colonies. The results were derived from three repeated experiments.

### Flow cytometry

Flow cytometric detection was carried out to evaluate the effects of secreted fractions from hypoxia- or normoxia-preconditioned ADSCs on the cell cycle and apoptosis of HUVECs. For the cell cycle analysis, the HUVECs were treated with solution H or N for 24 h, washed twice with sterile PBS, and fixed in 70% ice-cold ethanol. The mixture was stored at 4 °C overnight for further use. The HUVECs were treated with a mixture of 25 μl propidium iodide and 20 μl RNaseA at 37 °C for 0.5 h. The mixture was analyzed with flow cytometry (BD Biosciences Inc., NJ, USA). After the induction of apoptosis, the HUVECs were harvested and resuspended in 300 μl binding buffer supplemented with 5 μl Annexin V-FITC. The mixture was stored in the dark at 4 °C for 0.5 h, and then, 5 μl PI was added for 5 min. The outcome was analyzed with FACSDiva Software (BD Bioscience). The necrotic cells, late-stage apoptotic cells, early apoptotic cells, and live cells were identified as V-FITC−/PI+ (upper left quadrant), Annexin V-FITC+/PI+ (upper right quadrant), Annexin V-FITC+/PI− (lower right quadrant), and Annexin V-FITC−/PI− (lower left quadrant), respectively.

### Cell migration assay

To evaluate the effect of secreted fractions from hypoxia- or normoxia-preconditioned ADSCs on the migration of HUVECs, we performed wound healing assays and transwell migration assays.

For the wound healing assay, the HUVECs were seeded in a 6-well plate as a monolayer. A sterile 200-μl pipette tip was used to scratch a straight line. Then, solutions H or N were applied. Images were obtained at 24 h and analyzed. The results were derived from three repeated experiments.

For the transwell migration assay, 1.0 × 10^5^ cells were seeded in the upper chamber of transwell 24-well plates (Corning, USA). The plate included 8-μm pores for cell migration. After 12 h, the media in the lower chambers of the 24-well plates were replaced with solution H or N. After 12 h, the cells in the upper chamber were removed by a cotton swab, and the cells on the membrane were stained with 0.1% crystal violet solution (Sigma–Aldrich). The results were analyzed by ImageJ software.

### Angiogenic-related assessment

The levels of VEGF, hepatocyte growth factor (HGF), and fibroblast growth factor (FGF) that were secreted by hypoxia- or normoxia-preconditioned ADSCs were tested by enzyme-linked immunosorbent assay (ELISA). When the ADSCs reached 80–90% confluence, they were washed with sterile PBS and cultured under hypoxic (1% O_2_) or normoxic (21% O_2_) conditions. After 2 days of culture, the supernatant was concentrated and measured by ELISA kits (VEGF: ab100663, FGF: ab99979, and HGF: ab100534, Abcam, UK) according to the manufacturer’s instructions. The results were derived from three repeated experiments.

The expression of VEGFR2, the key receptor of VEGFA, was tested by Western blotting (WB) and quantitative real-time polymerase chain reaction (RT-qPCR). The HUVECs were cultured in solution H or N for 24 h and labeled as groups H and N. The cells were lysed with lysis buffer, and the protein concentration was measured with a BCA kit. Fifty micrograms of protein was acquired from groups H and N, resolved by SDS-PAGE, and transferred to PVDF membranes. After blocking with 5% skim milk, the membranes were incubated with primary antibodies against VEGFR2 (ab39638, Abcam). After overnight incubation at 4 °C, the membranes were incubated with secondary antibodies for 1 h. After incubation, the membranes were exposed and photographed. The data were analyzed by Quantity One software (Hercules). The mRNA of VEGFR2 was measured with RT-qPCR. After the cells were cultured with solution H or N for 24 h, the total RNA was extracted from the HUVECs by TRIzol Reagent (Invitrogen, USA) according to the manufacturer’s protocol. cDNA was synthesized using a Reverse Transcription Kit (Takara, Japan). The primers and cDNA were added to the SYBR Green PCR master mix (Takara, Japan) according to the manufacturer’s protocol. GAPDH was chosen as the internal control. All the primer sequences are listed in Table S1.

The angiogenic activity of hypoxia- or normoxia-preconditioned ADSCs was assessed by tube formation assay. A 24-well plate was precoated with Matrigel (BD Biosciences, San Jose, CA, USA) for 24 h. HUVECs (1.5 × 10^5^) were harvested and seeded in each well, cultured in solutions H or N, and labeled as groups H and N. After 10 h of incubation at 37 °C, capillary formation was observed by phase-contrast microscopy (Olympus, Japan). The outcome was analyzed by Image-Pro Plus 6.0 software (Media Cybernetics, Inc., MD, USA).

### Metabolic activity assays

The effect of hypoxia- or normoxia-preconditioned ADSCs on the metabolic activity of HUVECs was evaluated by measuring the mRNA and protein expression levels of hypoxia-inducible factor-1α (HIF-1α) (NB100-105, Novus) and hexokinase 2 (HK2) (Cell Signaling, Cst #2867). HUVECs were cultured in solution H or N for 24 h, which were labeled as groups H and N, and the total protein and mRNA were extracted and assessed by WB and RT-qPCR. The detailed procedure is described above.

### Transfection of siRNA

Small interfering RNA (siRNA) were transfected according to the manufacturer’s protocol (GenePharma Co, Shanghai, China). The ADSCs were cultured in FBS-free medium 30 min before the siRNA transfection. Then, 100 pmol of si-VEGF or si-Control was transiently transfected by Lipofectamine 2000. The medium was changed to complete medium 6 h later. The content of secreted VEGFA was measured by ELISA. si-VEGF or si-Control + hypoxia-preconditioned and si-VEGF or si-Control + normoxia-preconditioned ADSCs were used to treat HUVECs. The mRNA and protein expression levels of VEGFR2, HIF-1α, and HK2 were determined. The tube formation assay was performed to determine the angiogenic activity.

### Fabrication of porous, bilayered, nanofibrous scaffolds

The fabrication of the scaffold was previously described [25]. Briefly, the microporous inner layer was fabricated by a poly L-lactic acid (PLLA)/poly lactic-co-glycolic acid (PLGA)/poly L-lactide-co-e-caprolactone (PLCL) blend with a ratio of 20:20:60 using the thermally induced phase separation (TIPS) technique. Next, a PLLA/PLGA/poly caprolactone (PCL) blend with a ratio of 30:40:30 was cast onto the surface of the microporous inner layer. After separation, solvent exchange, and freeze-drying, the scaffold was obtained.

### Seeding ADSCs on scaffolds and identification

ADSCs were seeded on the scaffold and identified by scanning electron microscopy (SEM), immunohistochemistry (IHC), and confocal laser scanning microscopy (CLSM).

Before seeding, the scaffold was immersed in 75% alcohol for 48 h and washed 3 times with sterilized distilled water. Then, the scaffold was treated with ultraviolet rays overnight to prepare for the seeding of ADSCs. ADSCs at passage 3 and at 80–90% confluence were chosen as the seed cells. ADSCs (1.0 × 10^7^) were seeded on the outer layer of the scaffold. The scaffolds were left in culture for 3 days for identification and surgical implantation. The samples were fixed with 2.5% glutaraldehyde and dehydrated with an ascending series of ethanol. Then, the samples were dried in a vacuum and sputter-coated with gold for SEM observation (Hitachi TM-1000, Japan).

Before seeding the ADSCs on the scaffold, the ADSCs were transfected by virus to emit red fluorescence according to the manufacturer’s instructions (Hanbio, Shanghai, China). Briefly, 5 × 10^4^ ADSCs were seeded in each well of a 24-well plate. The multiplicity of infection (MOI) was 30. Puromycin (8 μg/ml) was used to select the infected ADSCs. ADSCs (1.0 × 10^7^/ml) were seeded on the outer layer of the scaffold (2 × 0.6 cm). The cells were stained with DAPI solution and washed with PBS. The organization and morphology of the fluorescent ADSCs on the scaffold were observed by confocal laser scanning microscopy (CLSM, Carl Zeiss, Germany). The cell-seeded scaffold was immersed in optimal cutting temperature (OCT) medium. Sections of 8 μm were generated with a freezing microtome (Thermo Scientific, USA), stained with a DAPI solution, and then observed by fluorescence microscopy (Nikon, Japan).

### Surgical procedure

A total of 15 New Zealand white rabbits were chosen for surgery and divided into 3 groups: the H, N, and Normal groups. The rabbits in the H or N group received scaffolds integrated with its own isolated hypoxia- or normoxia-preconditioned ADSCs, respectively. The rabbits in the Normal groups only received inguinal adipose tissue isolation, its urethra is intact. In H or N groups, 3 rabbits were randomly chosen at 3 or 6 months after implantation and then humanely euthanized to harvest the urethra. In Normal groups, all 3 rabbits were humanely euthanized to harvest the urethra at 6 months for comparison.

The implantation of the cell-seeded scaffolds was performed by the same experienced surgeon. All the New Zealand white rabbits were intravenously injected with pentobarbital sodium for general anesthesia. Then, an 8-Fr transurethral catheter was inserted into the urethra to support the urethra. After separating the rectum from the penis, a 2.0-cm ventral midline longitudinal incision was made to expose the urethra. On the ventral side, a 2.0 × 0.6 cm defect was made. The scaffolds integrated with hypoxia- or normoxia-preconditioned ADSCs were placed over the defect of the urethra. An interrupted suture was made to anastomose the edge of the scaffold and margin of the normal urethra with 6/0 vicryl sutures (Ethicon, USA). The corpus spongiosum was closed in a routine fashion. The inserted catheter was immobilized for bladder drainage, and ampicillin G (2 g/day) was given intravenously as an anti-inflammatory measure 3 days postoperatively. Any poor states, such as dysuria, energielos, and gastrointestinal symptoms, were closely observed.

### Retrograde contrast urethrography

Six months after the surgery, the rabbits receiving anterior urethroplasty were subjected to urethrography to determine the urethra caliber. Before urethrography, the rabbits were intravenously injected with pentobarbital sodium for general anesthesia. The rabbits were placed in the supine position. The catheter was inserted into the urethra and fixed with glans. The contrast medium, composed of Iobitridol (GE, China) mixed with normal saline at a 1:1 ratio, was injected into the catheter for the urethrogram. An X-ray machine (Philips, Netherlands) was used to capture the urethrography images.

### Morphological observation

Three or 6 months after the surgery, the rabbits were euthanized by a lethal pentobarbital injection, and the reconstructed urethra was harvested. The acquired tissues were immersed in paraformaldehyde overnight and transferred to 30% sucrose in water for 3 days. The tissue was sectioned at a thickness of 8 μm by a freezing microtome (Thermo Scientific, USA). Hematoxylin-eosin (H&E) staining and immunofluorescence (IHC) staining were performed to assess the morphology, urothelial cell layer (ab961, Abcam), smooth muscle cells (ab7817, Abcam), and vessel tissue (ab778, Abcam). The nuclei were stained with DAPI. Six random microscopic fields were chosen in each section to evaluate the urothelium, smooth muscle, and blood vessels. The results were analyzed by Image-Pro Plus 6.0 software (Media Cybernetics, USA).

### Statistical analysis

The data were analyzed by Statistics Package for Social Science (SPSS 24.0). *T* test analysis and one-way and two-way ANOVA were applied for comparisons between groups. The final data are reported as the mean ± standard deviation. *p* < 0.05 represents statistical significance.

## Results

### Schematic of the fabrication of the scaffold, in vivo and in vitro experiments

**Fig. 1:**
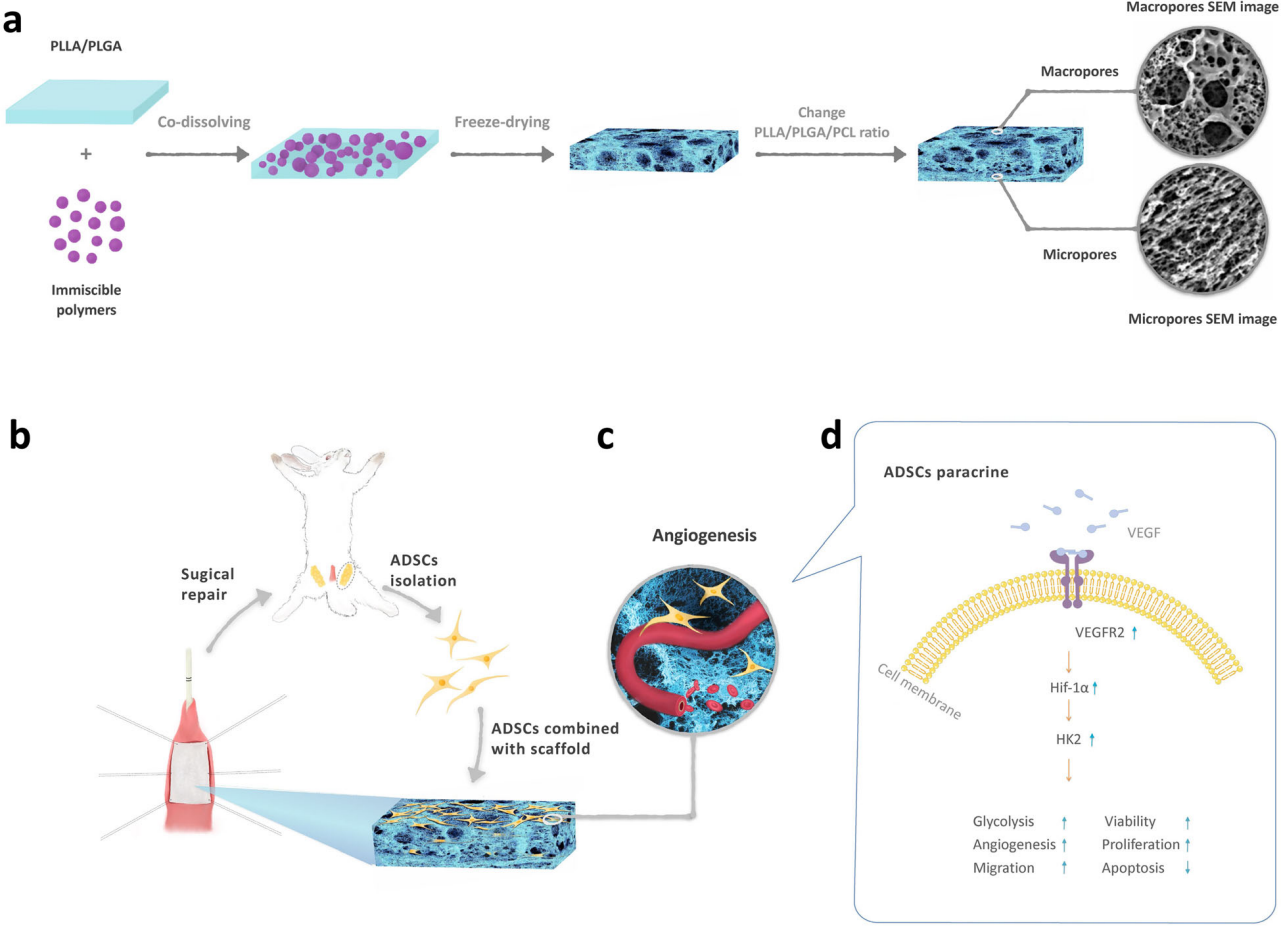
Schematic of the fabrication of the scaffold, in vivo and in vitro experiments. **a** Fabrication of the porous bilayered nanofibrous scaffold. **b** ADSCs isolated from the bilateral inguinal adipose tissues and then seeded to the scaffold to repair the defect of the urethra. **c** Hypoxia-preconditioned ADSCs grow in macropores and promote angiogenesis. **d** The mechanism of hypoxia-preconditioned ADSCs secreted more VEGFA to promote the expression of VEGFR2, HIF-1α, and HK2 to upregulating glycolysis

Figure 1a is a schematic illustration of the fabrication of the porous bilayered nanofibrous scaffold. Figure 1b depicts that we isolate ADSCs from the bilateral inguinal adipose tissues and then seeded them to the scaffold to repair the defect of the urethra. Figure 1c illustrates hypoxia-preconditioned ADSCs grow in macropores and promote angiogenesis. Figure 1d illustrates the mechanism of hypoxia-preconditioned ADSCs secreted more VEGFA to promote the expression of VEGFR2, HIF-1α, and HK2 to upregulating glycolysis.

### ADSC morphology and multipotency analysis

After isolation and culture, the ADSCs showed spindle-shaped, fibroblast-like morphology. ADSCs of passage 0–3 were photographed and are shown in Fig. S1. Differentiation media were used to analyze the multipotency of the ADSCs. The adipogenic, chondrogenic, and osteogenic lineages are shown in Fig. S2.

### Hypoxia-preconditioned ADSCs improve the proliferation and viability of HUVECs

**Fig. 2:**
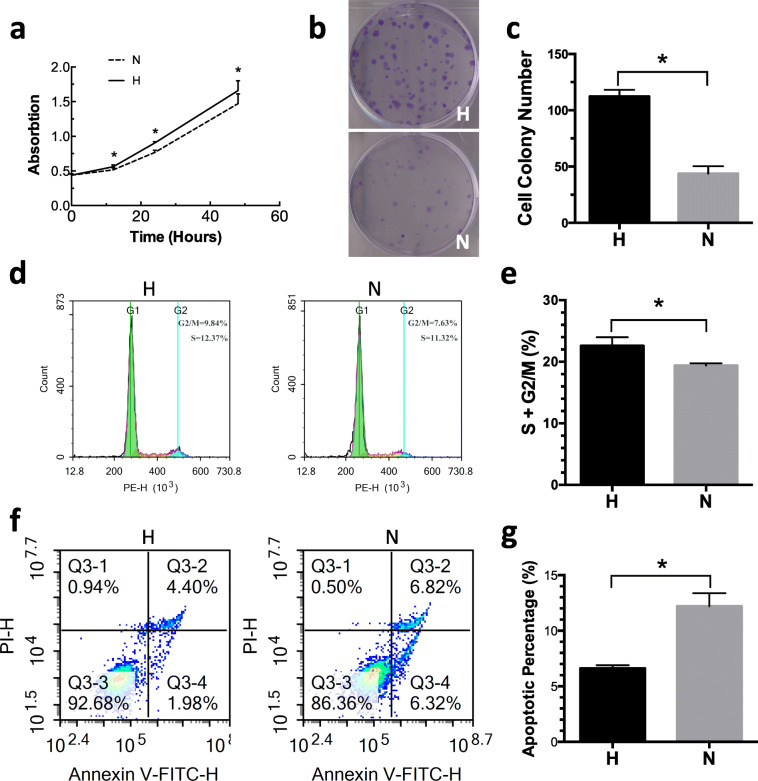
Role of hypoxia- or normoxia-preconditioned ADSCs in proliferation and apoptosis of HUVECs. **a** Proliferation of HUVECs influenced by secreted fractions from hypoxia- or normoxia-preconditioned ADSCs (H or N group) was evaluated with Cell Counting Kit-8 (CCK8) assay at 2, 12, 24, and 48 h after cell seeding. *n* = 6/group. 2, 12, 24, 48 h, *p < 0.05. **b** The outcome of colony formation assays formed by HUVECs influenced by secreted fractions from hypoxia- or normoxia-preconditioned ADSCs (H or N group). **c** Quantification of colonies formed from HUVECs. *p < 0.05. **d**, **e** Cell cycle assessment was tested by flow cytometry. HUVECs at S+G2/M phase was significantly increased after affected by secreted fractions from hypoxia-preconditioned ADSCs. *p < 0.05. **f**, **g** Cell apoptosis was determined by flow cytometry. Hypoxia-preconditioned ADSCs could significantly decrease the apoptotic percentage of HUVECs. *p < 0.05

To examine the possible effect of hypoxia-preconditioned ADSCs, CCK-8 assays, colony formation assays, and flow cytometry were performed. Based on the outcome of the CCK-8 assay, we found that the absorption of group H at 12, 24, and 48 h was significantly higher (0.56 ± 0.03 vs 0.52 ± 0.03, 0.90 ± 0.02 vs 0.77 ± 0.03, 1.66 ± 0.14 vs 1.43 ± 0.11 absorption, respectively, all *p* < 0.05) than that of group N (Fig. 2a). This indicates that hypoxia-preconditioned ADSCs could significantly improve the proliferation of HUVECs. The colony formation assays showed that the HUVECs treated with hypoxia-preconditioned ADSCs had improved colony-forming abilities and formed more colony units (112.33 ± 5.86 vs 43.67 ± 6.66, *p* < 0.05) (Fig. 2b, c). Cell cycling largely reflects cell behavior and proliferation. Based on the cell cycle analysis (Fig. 2d, e), we observed more HUVECs at the S + G2/M stage (22.62 ± 1.39 vs 19.38 ± 0.39%, *p* < 0.05) after treatment with hypoxia-preconditioned ADSCs. To investigate the anti-apoptotic role of hypoxia-preconditioned ADSCs, the HUVECs were stained with annexin V and measured by flow cytometry (Fig. 2f, g). The percentages of apoptotic HUVECs were significantly lower (6.61 ± 0.28 vs 12.21 ± 1.18%, *p* < 0.05) after treatment with hypoxia-preconditioned ADSCs. These results showed that compared to normoxia-preconditioned ADSCs, hypoxia-preconditioned ADSCs could improve the proliferation and viability of HUVECs.

### Hypoxia-preconditioned ADSCs improve the migration of HUVECs

**Fig. 3:**
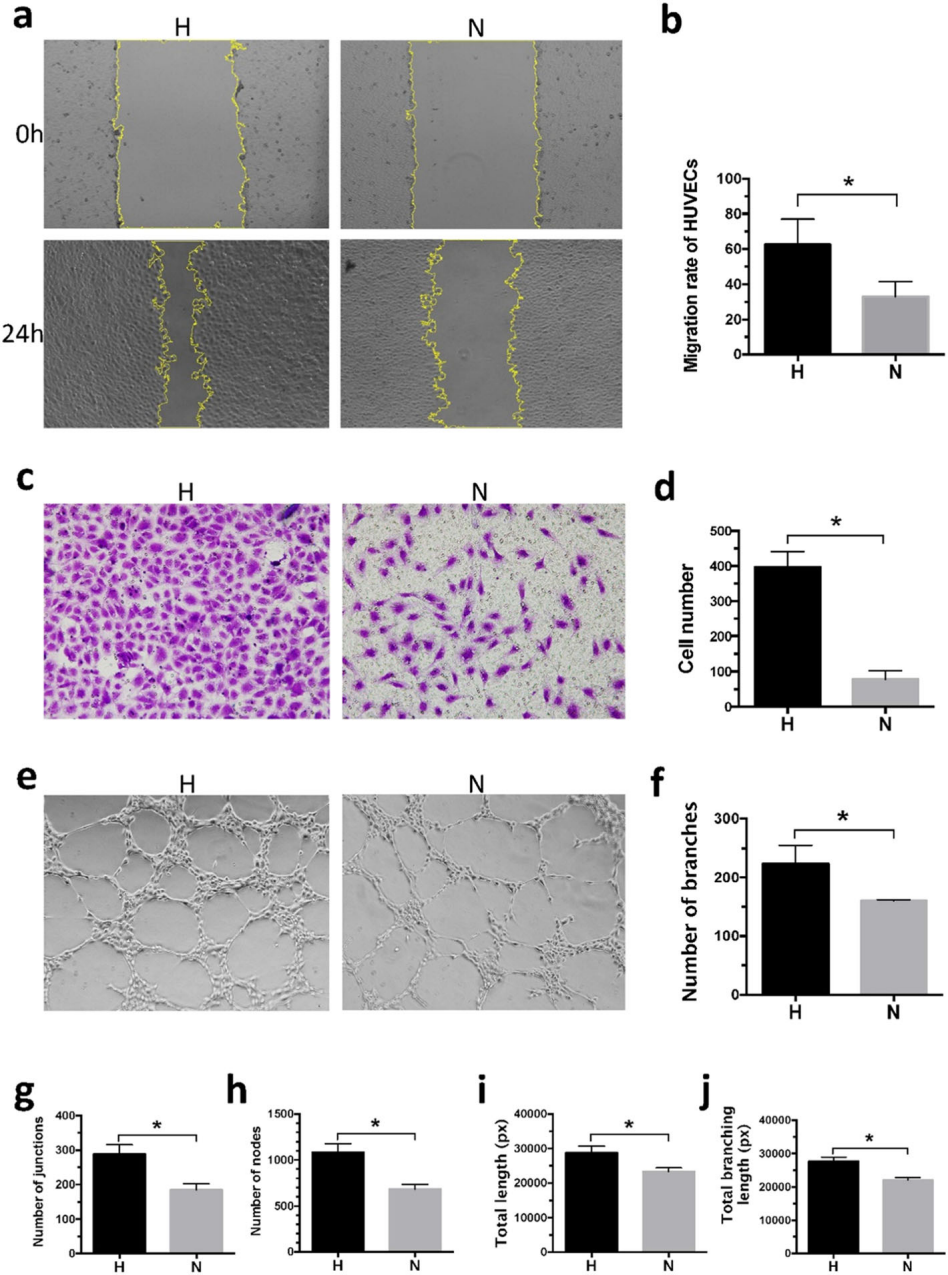
Role of hypoxia- or normoxia-preconditioned ADSCs in migration and angiogenesis of HUVECs. **a**, **b** Representative photos of scratch on HUVECs at 0 h and 24 h after treated by secreted fractions from hypoxia- or normoxia-preconditioned ADSCs. The migration of HUVECs was significantly increased by secreted fractions from hypoxia-preconditioned ADSCs. *p < 0.05. **c**, **d** Representative photos of transwell migration assay of HUVECs after treated by secreted fractions from hypoxia- or normoxia-preconditioned ADSCs. Hypoxia-preconditioned ADSCs increased migrated HUVECs. *p < 0.05. **e** In vitro tube formation of HUVECs at 10 h in different groups. **f**–**j** The quantification of branches, junctions, nodes, total length, and total branching length was listed and measured by Image-Pro Plus 6.0 (IPP). *p < 0.05

To determine the effect of hypoxia-preconditioned ADSCs on HUVEC migration, wound healing assays and transwell migration assays were performed. Hypoxia- or normoxia-preconditioned ADSCs both facilitated wound closure at 24 h. The percentage of the healed area at 24 h was significantly larger (62.57 ± 14.57 vs 33.08 ± 8.52%, *p* < 0.05) after treatment with secreted fractions from hypoxia-preconditioned ADSCs (Fig. 3a, b). The migration-promoting effect of hypoxia-preconditioned ADSCs was further confirmed by a transwell migration assay. Figure 3c and d show that hypoxia-preconditioned ADSCs significantly increased the number of migrated HUVECs (396.67 ± 43.75 vs 78.33 ± 24.01, *p* < 0.05). These outcomes implied that hypoxia-preconditioned ADSCs could dramatically promote the migration capacity of HUVECs.

### Hypoxia-preconditioned ADSCs improve angiogenesis in vitro

To evaluate the effect of hypoxia- or normoxia-preconditioned ADSCs on angiogenic capacity, tube formation assays were performed (Fig. 3e). The number of branches, junctions, and nodes; the total length; and the branching length were analyzed (223.67 ± 31.72 vs 160.00 ± 2.00, 288 ± 27.06 vs 185 ± 17.09, 1083.33 ± 94.48 vs 686.00 ± 46.70, 28,798 ± 1894.05 vs 23,352.67 ± 1021.19 px, 27,673.67 ± 1267.75 vs 21,980.33 ± 838.65 px, respectively, all *p* < 0.05, Fig. 3f–j). Our data showed that these features were significantly improved after hypoxia-preconditioned ADSC treatment compared with normoxia-preconditioned ADSC treatment. Therefore, compared to normoxia-preconditioned ADSCs, hypoxia-preconditioned ADSCs could facilitate angiogenesis in vitro.

### Hypoxia-preconditioned ADSCs upregulate VEGFA secretion

**Fig. 4:**
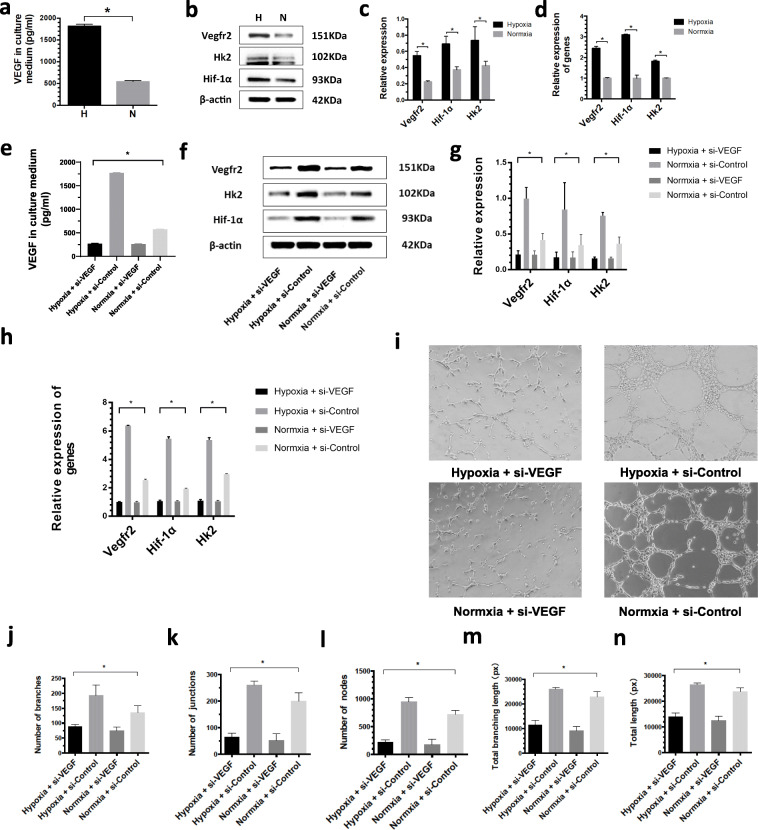
Role of hypoxia-preconditioned ADSCs in glycolysis of HUVECs. **a** VEGF secreted by hypoxia- or normoxia-preconditioned ADSCs determined by ELISA. *p < 0.05. **b**, **c** The protein expression of VEGFR2, Hif-1α, and HK2 determined by WB. *p < 0.05. **d** The transcription level of VEGFR2, Hif-1α, and HK2 determined by RT-qPCR. *p < 0.05. **e** Detection of VEGF after ADSCs were transfected by siRNA. *p < 0.05. **f**, **g** The protein expression of VEGFR2, Hif-1α, and HK2 after ADSCs transfected by siRNA were determined by WB. *p < 0.05. **h** The transcription level of VEGFR2, Hif-1α, and HK2 after ADSCs transfected by siRNA was determined by RT-qPCR. *p < 0.05. **i**–**n** The angiogenic activity of hypoxia- or normoxia-preconditioned ADSCs was assessed by tube formation assay. *p < 0.05

To evaluate whether hypoxia-preconditioned ADSCs secrete more VEGF, an ELISA assay was performed. Figure 4a shows that hypoxia-preconditioned ADSCs secreted more VEGFA than normoxia-preconditioned ADSCs (1814.92 ± 43.17 vs 541.05 ± 25.49, *p* < 0.05). There were no significant differences in the secreted HGF between hypoxia- and normoxia-preconditioned ADSCs (Fig. S3, *p* > 0.05). The FGF content was too low and could not be measured.

### Hypoxia-preconditioned ADSCs facilitate the metabolic activity of HUVECs

To examine the metabolic activity of HUVECs after treatment with secreted fractions from hypoxia- or normoxia-preconditioned ADSCs, the transcription and expression levels of VEGFR2, HIF-1α, and HK2 were examined. HIF-1α and HK2 play vital roles in glycolysis [28]. The results showed that hypoxia-preconditioned ADSCs significantly upregulated the transcription of VEGFR2, HIF-1α, and HK2 (2.45 ± 0.09 vs 1.00 ± 0.03, 3.10 ± 0.04 vs 1.00 ± 0.16, 1.81 ± 0.06 vs 1.00 ± 0.02, respectively, all *p* < 0.05, Fig. 4d). Hypoxia-preconditioned ADSCs also significantly upregulated the protein expression of VEGFR2, HIF-1α, and HK2 compared to normoxia-preconditioned ADSCs (0.55 ± 0.05 vs 0.23 ± 0.01, 0.69 ± 0.10 vs 0.37 ± 0.04, 0.73 ± 0.17 vs 0.42 ± 0.06, all *p* < 0.05, Fig. 4b, c). These results suggested that hypoxia-preconditioned ADSCs could improve the metabolic activity of HUVECs.

### Hypoxia-preconditioned ADSCs promote the metabolic activity and angiogenesis of HUVECs through VEGFA in vitro

To determine what factors mediate the effects of ADSCs on promoting HUVEC metabolic activity and angiogenesis in vitro, we silenced the expressing of VEGFA. After silencing the expressing of VEGFA, we measured the content of VEGFA in the medium from hypoxia- or normoxia-preconditioned ADSCs. The results showed that the transfection of si-VEGF significantly suppressed VEGFA secretion into the medium (268.42 ± 10.99 vs 1770.25 ± 10.04 vs 259.12 ± 10.76 vs 574.24 ± 6.23, Fig. 4e). HUVECs were treated with si-VEGF or si-Control + hypoxia-preconditioned ADSCs and si-VEGF or si-Control + normoxia-preconditioned ADSCs, and the transcription and expression levels of VEGFR2, HIF-1α, and HK2 were examined. The protein levels were significantly suppressed by si-VEGF + hypoxia-preconditioned ADSCs (VEGFR2, 0.21 ± 0.05 vs 1.00 ± 0.16 vs 0.21 ± 0.05 vs 0.42 ± 0.09; HIF-1α, 0.17 ± 0.07 vs 0.84 ± 0.38 vs 0.17 ± 0.08 vs 0.34 ± 0.15; HK2, 0.16 ± 0.02 vs 0.76 ± 0.05 vs 0.16 ± 0.02 vs 0.36 ± 0.10, Fig. 4f, g). The mRNA results were identical (VEGFR2, 1.01 ± 0.02 vs 6.38 ± 0.03 vs 0.99 ± 0.06 vs 2.55 ± 0.07; HIF-1α, 1.07 ± 0.06 vs 5.46 ± 0.14 vs 1.06 ± 0.05 vs 1.93 ± 0.05; HK2, 1.09 ± 0.09 vs 5.37 ± 0.18 vs 1.06 ± 0.07 vs 2.97 ± 0.04, Fig. 4h). Angiogenesis was also inhibited by si-VEGF + hypoxia-preconditioned ADSCs (Fig. 4i). The branches, junctions, nodes, total branching length, and total length were significantly decreased after treatment with si-VEGF + hypoxia-preconditioned ADSCs (branches, 89.00 ± 6.00 vs 193.00 ± 34.83 vs 75.67 ± 11.59 vs 135.00 ± 23.39; junctions, 64.33 ± 14.29 vs 260.67 ± 15.28 vs 49.67 ± 28.02 vs 200.67 ± 30.37; nodes, 219.33 ± 41.79 vs 947.33 ± 75.06 vs 183.67 ± 86.50 vs 721.00 ± 73.57; total branching length, 11,463.67 ± 1885.01 px vs 25,987.67 ± 627.71 px vs 9220.00 ± 1564.82 px vs 23,032.33 ± 1933.45 px; total length, 13,948.67 ± 1452.14 px vs 26,428 ± 755.52 px vs 12,480.67 ± 1764.79 px vs 23,716.33 ± 1507.00 px, Fig. 4j–n).

### Fabrication of the scaffold and combining cells with scaffold

**Fig. 5:**
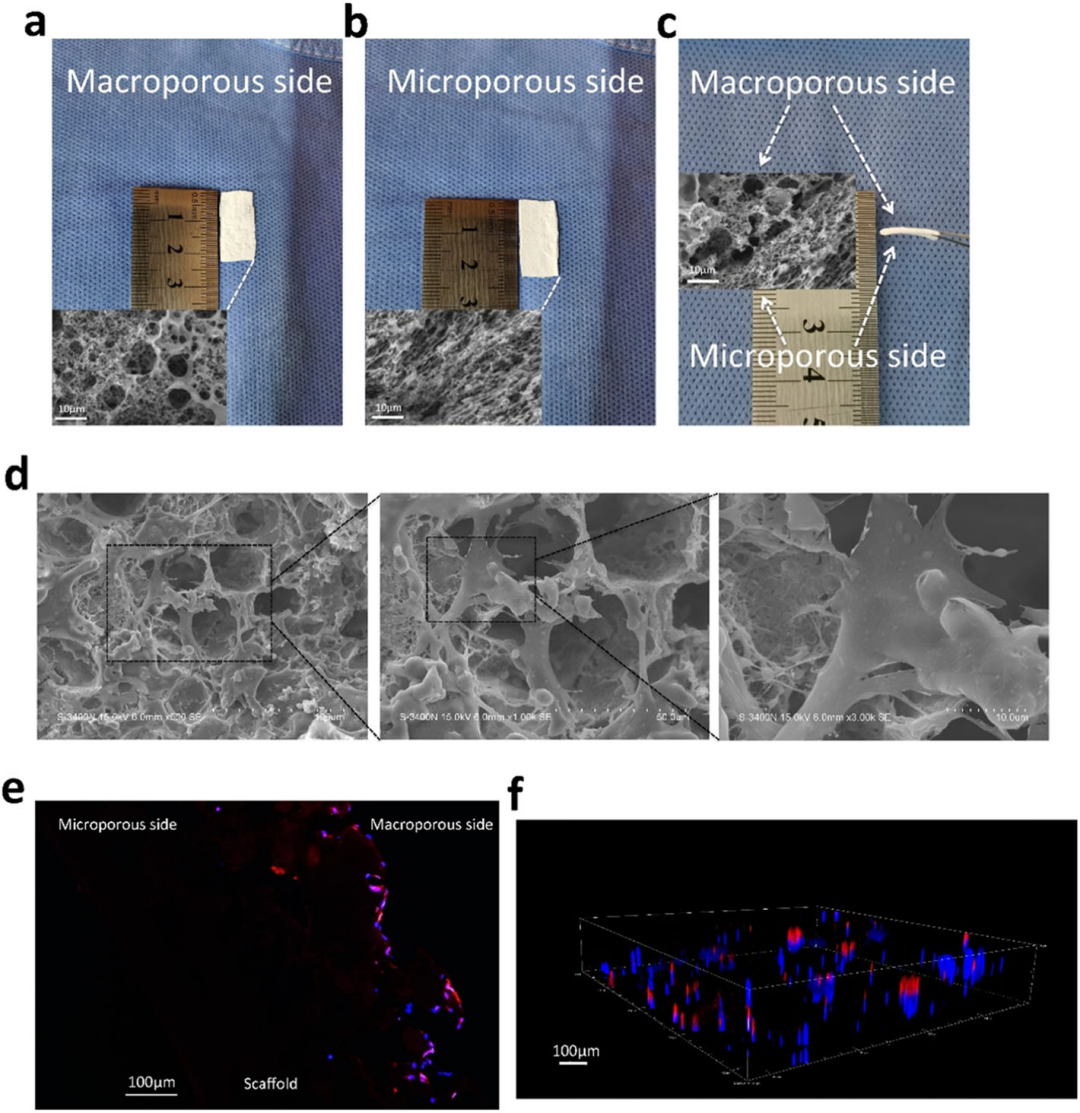
The morphology of the scaffold and identification of ADSCs seeded on scaffold. **a**–**c** Macroporous, microporous, and cross-section sides of the scaffolds. The morphology of the scaffold was detected by SEM. Scale bar = 10 μm. **d** The HUVECs were attached with the pore of the scaffold and detected by SEM. Scale bar = 100 μm. **e** The immunofluorescence section of the scaffold was observed by a fluorescent microscope. Scale bar = 100 μm. **f** The organization and morphology of fluorescent ADSCs on the scaffold were observed by a confocal laser scanning microscope. Scale bar = 100 μm

The gross morphology and SEM observations of the macroporous side, the microporous side, and the interface of the scaffold are shown in Fig. 5a–c. The macroporous side was densely covered with large enough pores to allow the infiltration of cells and interstitial fluid flow. The microporous side could serve as a barrier to resist urine and provide an ideal environment for the growth of epithelial cells [29, 30]. Figure 5d shows SEM images of ADSCs cultured on the scaffold. The ADSCs perfectly combined with the scaffold and permeated into the macropores, showing irregularly spread morphology with elongated pseudopodia. To further show that HUVECs successfully combined and grew into the scaffold, IHC and CLMS were performed. Figure 5e shows the transfected ADSCs emitting red fluorescence and located in the macropores of the scaffold. The ADSCs were stained with DAPI. Figure 5f shows the three-dimensional microstructure of the ADSCs and scaffolds. The *z* axis of the CLSM image with a length of more than 75 μm indicates that ADSCs were successfully located in the macropores of the scaffold. The above results showed that the scaffold successfully combined with the cells and that the pores were suitable for neotissue growth and infiltration.

### Hypoxia-preconditioned ADSCs lead to fewer complications and a larger urethral caliber

**Fig. 6:**
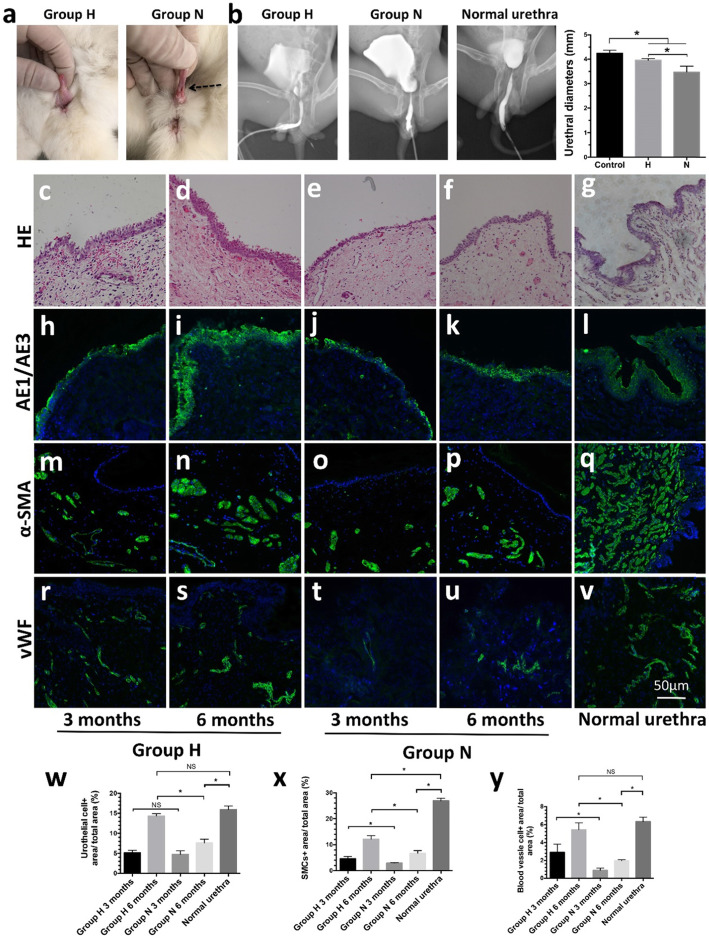
Postoperative complications, urethra caliber diameters, and morphological observation of the neourethra. **a** Relatively normal appearance of group H and fistula of group N. **b** Retrograde contrast urethrography of group H, N, and normal urethra. The diameter of the urethra in group H was significantly larger than that in group N. *p < 0.05. **c**–**g** Hematoxylin-eosin (H&E) staining image of urethra 3 and 6 months postoperatively. The epithelium (**h**–**l**), smooth muscle (**m**–**q**), and blood vessels (**r**–**v**) were evaluated by AE1/AE3, α-SMA, and vWF. Scale bar = 50 μm. **w**–**y** Image analysis for the contents of the epithelium, smooth muscle, and blood vessels in different groups. More epithelium, smooth muscle, and blood vessels were observed in group H than N. *p < 0.05, NS means *p* > 0.05

All rabbits survived the operation in which ADSCs combined with scaffolds were implanted. The surgical wounds healed 14 days postoperatively. Among the rabbits that received hypoxia-preconditioned ADSCs combined with scaffolds (group H), four rabbits did not present with symptoms of dysuria, and only two rabbits presented mild dysuria that had no significant impact on survival. In group N, after the removal of the urethral catheter, four of the rabbits presented symptoms of dysuria, such as thinner urine stream and distention of the bladder. One rabbit in group N presented urethral fistulas, and the urine transmitted out of the fistula (Fig. 6a). All the rabbits survived until 3 or 6 months after implantation. Retrograde urethrography showed that the diameters of the urethras in group H were larger than those in group N (*p* < 0.05). However, the diameters of the normal urethras were wider than those in groups H and N (4.24 ± 0.13 vs 3.96 ± 0.07 vs 3.47 ± 0.24 mm, *p* < 0.05, Fig. 6b).

### Hypoxia-preconditioned ADSCs facilitate neotissue regeneration in vivo

The regeneration of urothelial cells was confirmed by hematoxylin-eosin (H&E) staining (Fig. 6c–g) and immunofluorescence (IHC) staining (Fig. 6h–l). Compared with those in group N, in group H, the urothelial cells were arranged in a more orderly manner and in thicker urothelial cell layers at 3 and 6 months (*p* < 0.05). The regenerated urothelial cells in group H at 6 months showed stratification comparable to that of the normal urothelial cell layers (*p* > 0.05). The regenerated epithelial cells in group N were loosely arranged and thinner than those in group H and in the normal urothelium (Fig. 6w, group H 3 months vs group H 6 months vs group N 3 months vs group N 6 months vs normal urethra, 4.87 ± 1.56 vs 16.16 ± 3.30 vs 4.66 ± 1.33 vs 8.37 ± 1.06 vs 17.39 ± 2.94%, respectively). The smooth muscle cells (SMCs) were identified by the emission of bright green fluorescence (Fig. 6m–q) and HE staining (Fig. 6c–g). The SMC density of group H at 6 months was significantly increased compared with that of group N at 3 months (*p* < 0.05), and it was also significantly higher than that of group N at 3 or 6 months (*p* < 0.05). However, the SMC content of group H at 6 months was still significantly lower than that of the normal urethra (*p* < 0.05) (Fig. 6x, group H 3 months vs group H 6 months vs group N 3 months vs group N 6 months vs normal urethra, 6.68 ± 2.16 vs 15.00 ± 2.45 vs 3.71 ± 0.76 vs 7.08 ± 1.77 vs 22.60 ± 2.74%, respectively). The regenerated vessel tissue was stained and emitted bright green fluorescence (Fig. 6r–v). At 3 months, there was significantly more regenerated blood vessel tissue in group H than in group N (*p* < 0.05), showing the role of hypoxia-preconditioned ADSCs in angiogenesis. The regenerated blood vessel tissue of group H at 6 months was well organized, comparable to the normal urethra (*p* > 0.05) and significantly more well organized than that of group N (*p* < 0.05) (Fig. 6y, group H 3 months vs group H 6 months vs group N 3 months vs group N 6 months vs normal urethra, 5.15 ± 1.11 vs 7.05 ± 0.62 vs 3.40 ± 0.59 vs 5.04 ± 0.66 vs 7.34 ± 1.19%, respectively).

## Discussion

Our study evaluated the use of hypoxia- vs. normoxia-preconditioned ADSCs combined with a novel porous scaffold for anterior urethroplasty. The key finding is that hypoxia-preconditioned ADSCs facilitate the viability, migration, and angiogenesis of blood endothelial cells (ECs) for tissue regeneration, probably via increased glycolysis.

The identification of the appropriate hypoxic culture conditions is of great significance. Determining the ideal oxygen tension is the key to exploring the optimal culture conditions. ADSCs have been reported to reside in a lower (i.e., 1–5% O_2_) oxygen tension than that typically used in ambient cell culture (20–21%) [5]. Due to the lack of vascularization, in the early period of tissue regeneration, the oxygen tension may be lower than 4% [31]. Therefore, we used 1% O_2_ oxygen tension to simulate the oxygen conditions after cell-scaffold transplantation. Zhang et al. found that after hypoxia preconditioning, ADSCs secreted more angiogenic factors. Angiogenesis and histological injury were both significantly improved, and renal function was protected [32]. Valorani et al. revealed that compared to normoxic conditions, hypoxic conditions could enhance the migration and viability of ADSCs [33]. Stubbs et al. reported that after preconditioning with simulated hypoxia and ischemia conditions, ADSCs exhibited increased viability and reduced cell injury. The media of hypoxia-preconditioned ADSCs could enhance endothelial cell survival and angiogenesis capacity in vitro [34]. Our in vitro CCK-8, colony formation and flow cytometry data revealed that hypoxia-preconditioned ADSCs exhibited better function in promoting cell proliferation. These results are consistent with previous reports [33]. Subsequent tests showed that compared to normoxia-preconditioned ADSCs, hypoxia-preconditioned ADSCs could improve the migration of HUVECs. This finding is identical to previous results showing that conditioned medium derived from hypoxia-preconditioned ADSCs could enhance the migration of primary gastric mucosal epithelial cells (GECs) in vitro [35]. In addition, hypoxia-preconditioned ADSCs lead to greater angiogenic effects because they secrete more VEGFA and promote endothelial tube formation. This observation conforms to our results revealing that the increased paracrine production of VEGF-A by hypoxia-preconditioned ADSCs leads to a significantly diminished angiogenic response [17].

In our study, VEGFA and VEGFR2 were chosen as the main mediators of the effect of ADSCs on HUVECs. VEGFA is one of the most important vascularization-related factors and plays a vital role in vascularization and tissue engineering [23]. It has also been reported that VEGFA can increase glycolysis and ensure physiological metabolism [20, 36]. There is consensus that VEGFR-2 is the main receptor of VEGFA that plays an angiogenic role via tyrosine phosphorylation [31]. Therefore, we believe that in the interaction between ADSCs and HUVECs, VEGFA and VEGFR2 may represent two targeted molecules. The HIF-1 pathway plays a leading role in angiogenesis, glycolysis, and the hypoxic response. The stabilization of HIF-1α, the master glycolytic regulator, can upregulate glycolysis to promote cell viability and proliferation [37]. HIF-1α can combine with HIF-1β to bind to the hypoxia response element (HRE) in the cellular nucleus to regulate or reprogram genes related to glycolysis, such as HK2, which is the first rate-limiting enzyme in glycolysis [28]. As shown in our results, the cytokines secreted by hypoxia-preconditioned ADSCs could stimulate the HIF-1α-HK2 axis. The WB and RT-PCR results showed that HIF-1α and HK2 were both significantly upregulated. The angiogenesis function was enhanced, as shown by the tube formation test. After silencing VEGF expression in ADSCs, there was a marked decrease in the VEGF content. VEGFR-2, the key angiogenic receptor [38], was also downregulated. The WB and RT-PCR results showed that HIF-1α and HK2 were both significantly downregulated. The tube formation test showed that the angiogenic function of HUVECs was suppressed.

The phenomenon of the reliance of vessel endothelial cells on glycolysis is counterintuitive because endothelial cells are in close contact with oxygen in the blood, and one unit of glucose can produce 20-fold more ATP by the TCA cycle than by glycolysis [39]. In fact, less than 1% glucose in ECs enters the TCA cycle, and oxidative phosphorylation generates only 10% of all the ATP in ECs [22]. This phenomenon could be explained by various reasons. First, the levels of oxygen and glucose are both low in avascular tissues, but glucose diffuses further than oxygen. When endothelial cells sprout into avascular tissues, they can rely on glucose rather than oxygen to form vessels, as long as glucose is not limiting [40, 41]. Second, endothelial cells consume little oxygen by glycolysis, which can save more oxygen for perivascular cells. Third, compared to the tricarboxylic acid (TCA) cycle of oxidative metabolism, glycolysis can generate ATP more rapidly to supply energy during sprouting. Tip cells extend lamellipodia and filopodia to migrate. The formation of these motile structures relies on the remodeling of the actin cytoskeleton, which is a process that requires the rapid production of high amounts of ATP. Fourth, endothelial cells can reduce the harm of oxidation by minimizing the level of oxidative metabolism. Finally, the substrate for the macromolecules generated by glycolysis is essential for cell duplication and division [22].

Urethral defects or strictures affect nearly 1% of the male population and severely disrupt physical and mental conditions of these patients [42]. In the last two decades, the best tissue for use in urethral repair is the buccal mucosa [43]. However, obtaining the buccal mucosa may cause donor site morbidity. In these cases, tissue engineering techniques represent an alternative material for urethroplasty. These techniques can avoid the complications related to graft harvesting at the buccal mucosa site and provide a convenient and feasible substitution for reconstruction [43]. Cells combined with scaffolds are innovative methods to repair and regenerate the urethra. Liu et al. isolated urine-derived stem cells and seeded them onto the small intestinal submucosa (SIS) to repair the ventral side of the penile urethra in a rabbit model and achieved a satisfactory outcome [44]. Li et al. harvested and expanded rabbit bone mesenchymal stem cells (BMSCs) and smooth muscle cells (SMCs) and seeded them onto a bladder acellular matrix (BAM). After 2 weeks of implantation in the rabbit omentum, the graft was transplanted. Compared to cells combined with BAM, an acellular BAM showed a significantly higher rate of urethral strictures [45]. Filippo et al. compared collagen matrices with or without endothelial cells and SMCs. It was revealed that collagen matrices without cells lead to poor tissue development and stricture formation [46]. In our study, scaffolds combined with hypoxia-conditioned ADSCs accelerated the growth of vascular, muscle, and epithelial cells, making the structure closer to normal tissue. These scaffolds gain a better therapeutic effect with a larger diameter of the urethra lumen.

The scaffold serves as a temporary ECM for the proliferation and attachment of cells and plays a critical role in tissue regeneration [47]. Poly lactic-co-glycolic acid (PLGA) has good biodegradability and biocompatibility and is widely used in tissue engineering. PLGA degrades more rapidly and can be miscible with PLLA/PCL to accelerate the degradation of scaffolds [48]. A highly porous scaffold can provide the best environment for oxygen exchange, nutrient transport, and cell infiltration and then promote the growth of neotissues [49]. However, organs with lumens, such as the urethra and blood vessels, consist of layered cells and need heterogeneous porous structures for cell growth and tissue engineering applications [25]. The heterogeneous macroporous, nanofibrous side of the scaffold facilitates the infiltration of SMCs and vessel ECs, and the heterogeneous microporous luminal surface facilitates the growth of surrounding urethral epithelial cells [25]. The seeded ADSCs accelerate angiogenesis to supply oxygen and nutrients to urethral epithelial cells. The newly formed epithelium and the microporous luminal surface can protect the seeded cells and neotissues from urine, which could lead to urethral shrinkage, stricture, and obstruction. Therefore, the outer layer and inner layer complement each other and promote each other’s function. The interconnected heterogeneous pores (> 120 μm) closely mimic the pores of the ECM, and the average fiber diameter is similar to that of natural ECM fibers. In addition, it has been reported that the therapeutic ability of stem cells could be enhanced by grafts that support cell-cell interactions [6]. Therefore, the cells seeded on the macroporous side of our scaffold may enhance the paracrine ability due to increased cell-cell interactions.

Our study has several limitations. First, due to the limitations of the current technology, it is difficult to measure the exact oxygen concentration during the tissue engineering-mediated repair and regeneration of new tissue. Second, our animal experiments were conducted in normal animals, which cannot completely imitate the urethral conditions observed in the clinic. In the next phase, a new urethral model with a fibrotic urethral bed needs to be developed. Finally, the sexual function of the rabbits also needs to be tested, so the technology and equipment for detecting sexual function in rabbits need to be renewed.

## Conclusions

In summary, our study showed that hypoxia-preconditioned ADSCs combined with the newly fabricated PLLA/PCL/PLGA scaffold may offer innovative strategies for urethral reconstruction. In vitro, hypoxia-preconditioned ADSCs could secrete more VEGFA to promote the viability, proliferation, migration, and angiogenesis of blood ECs by increasing glycolysis. In vivo, the implantation of hypoxia-preconditioned ADSCs combined with scaffolds achieved better morphological preservation and revascularization. Due to the convenience of hypoxia treatment and the lack of genetic modification, hypoxia-preconditioned ADSCs combined with novel scaffold may be a promising alternative treatment for patients suffering from urethral defects or strictures in the clinic.

## Supplementary Information

**Additional file 1.** Supplementary figures and table

## Data Availability

The datasets used and/or analyzed during the current study are available from the corresponding author on reasonable request.

## Abbreviations

ADSCs: Adipose-derived stem cells
HUVECs: Human umbilical vein endothelial cells
ELISA: Enzyme-linked immunosorbent assay
VEGF-A: Vascular endothelial growth factor-A
ATP: Adenosine triphosphate
ECM: Extracellular matrix
H&E: Hematoxylin and eosin
α-MEM: α-Modified Eagle’s medium
FBS: Fetal bovine serum
CCK-8: Cell Counting Kit-8
FGF: Fibroblast growth factor
HGF: Hepatocyte growth factor
WB: Western blotting
RT-qPCR: Quantitative real-time polymerase chain reaction
HIF-1α: Hypoxia-inducible factor-1α
HK2: Hexokinase 2
siRNA: Small interfering RNA
TIPS: Thermally induced phase separation
SEM: Scanning electron microscopy
IHC: Immunohistochemistry
CLSM: Confocal laser scanning microscopy
SPSS: Statistics Package for Social Science
IPP: Image-Pro Plus
SMCs: Smooth muscle cells
GECs: Gastric mucosal epithelial cells
HRE: Hypoxia response element
TCA: Tricarboxylic acid
SIS: Small intestinal submucosa
BMSCs: Bone mesenchymal stem cells
BAM: Bladder acellular matrix
PLGA: Poly lactic-co-glycolic acid
PLLA: Poly l-lactic acid
PCL: Poly caprolactone
ECs: Endothelial cells
