# Supplementary material for: Hypoxia-preconditioned adipose-derived stem cells combined with scaffold promote urethral reconstruction by upregulation of angiogenesis and glycolysis
Source: Stem Cell Res Ther. 2020 Dec 11;11:535. doi: 10.1186/s13287-020-02052-4 (PMC7731784; doi:10.1186/s13287-020-02052-4)
Supplement: Supplementary file 1 — Additional file 1. Supplementary figures and table [file 13287_2020_2052_MOESM1_ESM.docx]

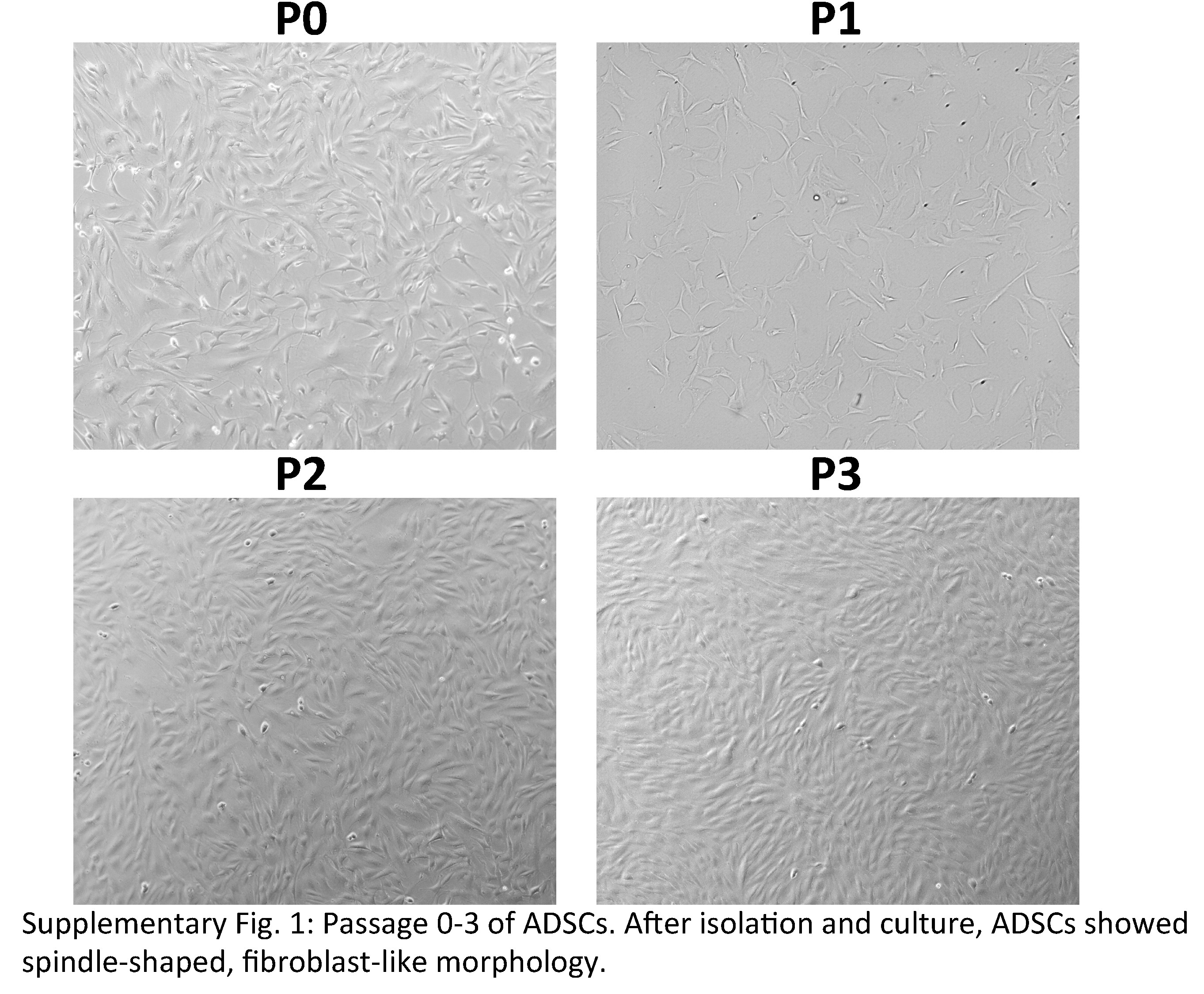


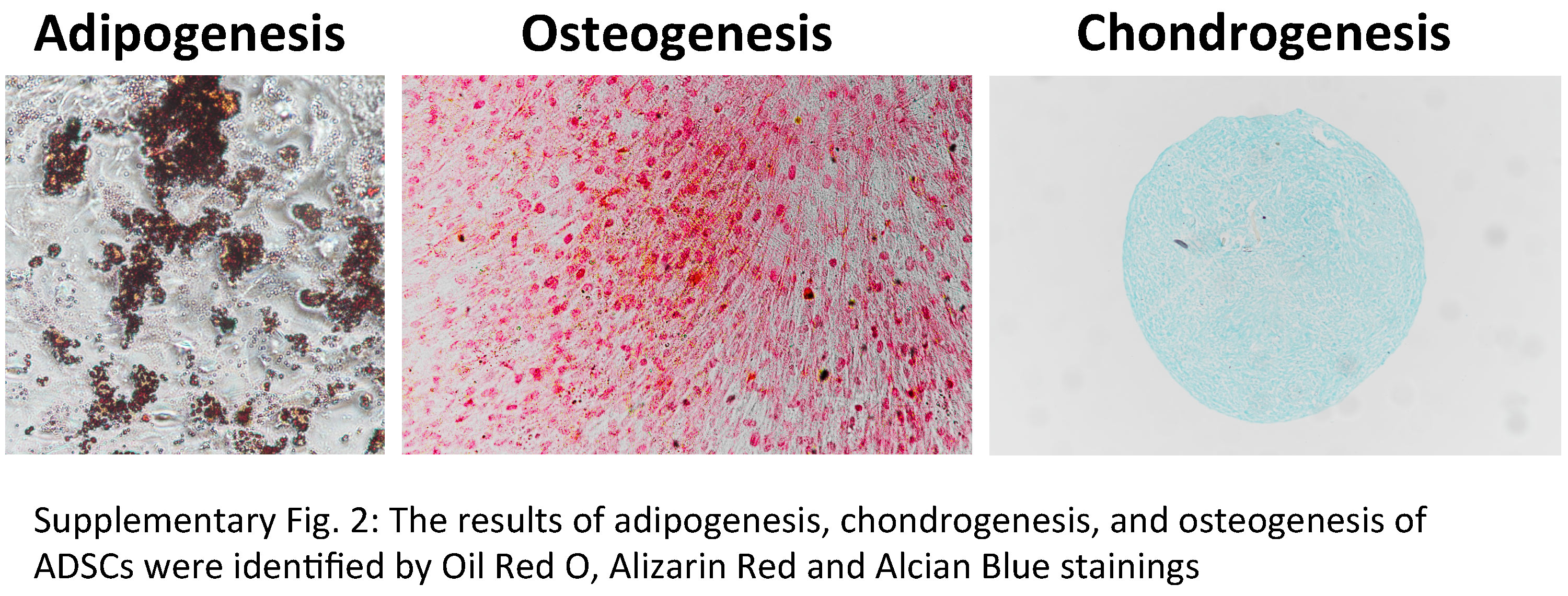


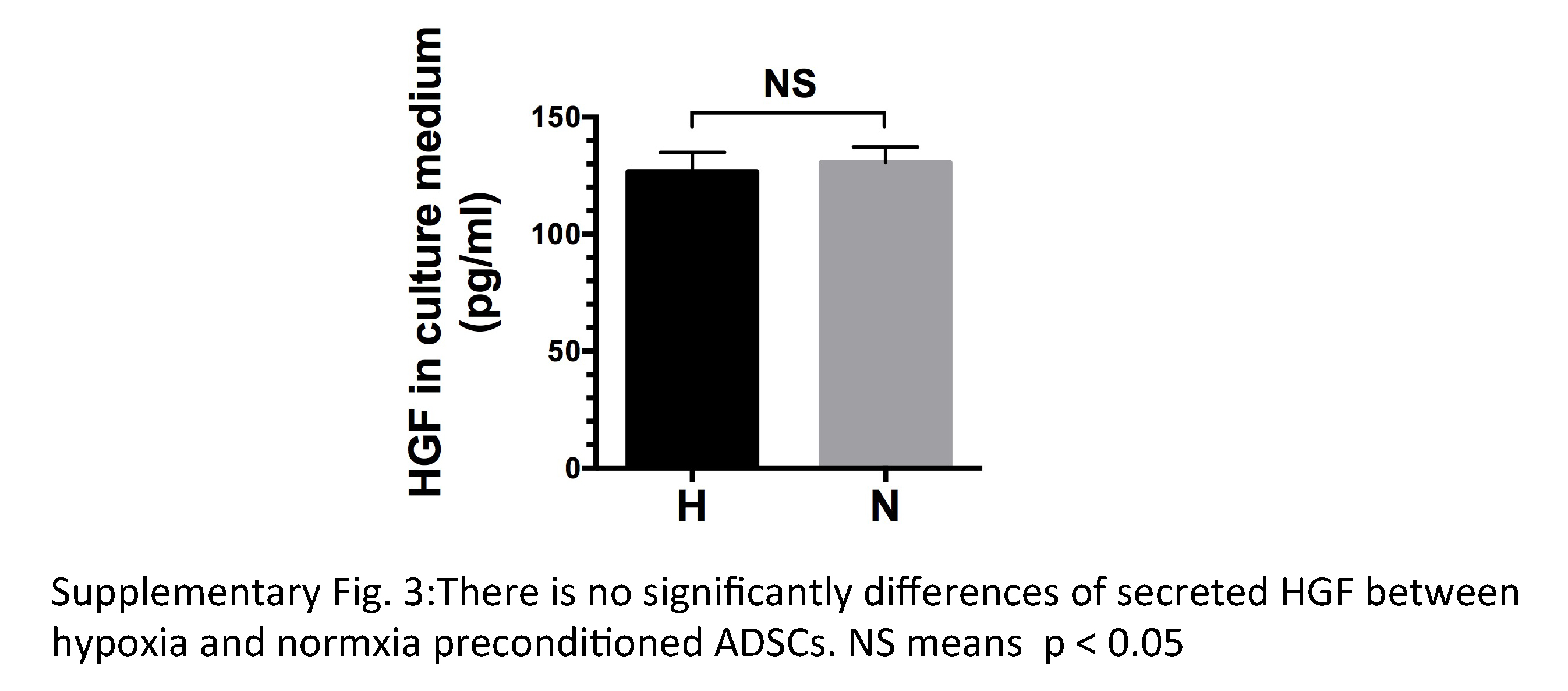


Supplementary table1: List of genes, primers and parameters used for PCR.

| Gene | Primer pairs | TM(℃) | Product size (bp) |
| --- | --- | --- | --- |
| Human HK2 | 5' ACT GGA CTG GGT TTT GTC TC 3' | 81.3 | 167 |
|  | 5' GGC AAG GGG GAT TAC TAA G 3' |  |  |
| Human VEGFR2 | 5' CAC CAC TCA AAC GCT GAC AT 3' | 83.3 | 82 |
|  | 5' CTC TTC CTC CAA CTG CCA ATA C 3' |  |  |
| Human Hif-1α | 5' ATG CTT ACA CAC AGA AAT GG 3' | 81.8 | 190 |
|  | 5' ACT GAG GTT GGT TAC TGT TG 3' |  |  |
| Human β-actin | 5' AAG GTG ACA GCA GTC GGT T 3' | 84.3 | 195 |
|  | 5' TGT GTG GAC TTG GGA GAG G 3' |  |  |
